# Design and synthesis of quasi-diastereomeric molecules with unchanging central, regenerating axial and switchable helical chirality via cleavage and formation of Ni(II)–O and Ni(II)–N coordination bonds

**DOI:** 10.3762/bjoc.8.223

**Published:** 2012-11-13

**Authors:** Vadim A Soloshonok, José Luis Aceña, Hisanori Ueki, Jianlin Han

**Affiliations:** 1 Department of Organic Chemistry I, Faculty of Chemistry, University of the Basque Country, 20018 San Sebastián, Spain; 2 IKERBASQUE, Basque Foundation for Science, 48011 Bilbao, Spain; 3 International Center for Materials Nanoarchitectonics (MANA), National Institute for Materials Science (NIMS), 1-1, Namiki, Tsukuba, Ibaraki 305-0044, Japan; 4 School of Chemistry and Chemical Engineering, Nanjing University, Nanjing, 210093, China

**Keywords:** axial chirality, central chirality, chiral switches, coordination bonds, functional materials, helical chirality, modular structural design, molecular devices

## Abstract

We describe herein the design and synthesis of asymmetric, pentadentate ligands, which are able to coordinate to Ni(II) cations leading to quasi-diastereomeric complexes displaying two new elements of chirality: stereogenic axis and helix along with configurational stabilization of the stereogenic center on the nitrogen. Due to the stereocongested structural characteristics of the corresponding Ni(II) complexes, the formation of quasi-diastereomeric products is highly stereoselective providing formation of only two, (*R*_a_*,*M*_h_*,*R*_c_*) and (*R*_a_*,*P*_h_*,*R*_c_*), out of the four possible stereochemical combinations. The reversible quasi-diastereomeric transformation between the products (*R*_a_*,*M*_h_*,*R*_c_*) and (*R*_a_*,*P*_h_*,*R*_c_*) occurs by intramolecular trans-coordination of Ni–NH and Ni–O bonds providing a basis for a chiral switch model.

## Introduction

The design and synthesis of organic molecules for which the changing of their three-dimensional structure or function can be predicted, is a fast-growing multidisciplinary field of research [1–13]. In particular, organic compounds that can undergo reversible diastereomeric transformations are considered as the most promising models for the development of molecular switches with nondestructive read out of the optical information [14–18]. Taking into account the issue of fatigue resistance or durability of a potential molecular switch, the newly emerging design strategies make use of the formation and cleavage of metal–ligand coordination bonds as a basis for a chiral conformational switch in the conformationally restricted molecular backbone [19–23]. Here, we would like to describe a new design and synthesis of quasi-diastereomeric molecules with unchanging central, regenerating axial, and switchable helical chirality, through cleavage/formation of Ni(II)–O and Ni(II)–N coordination bonds.

**Scheme 1:**
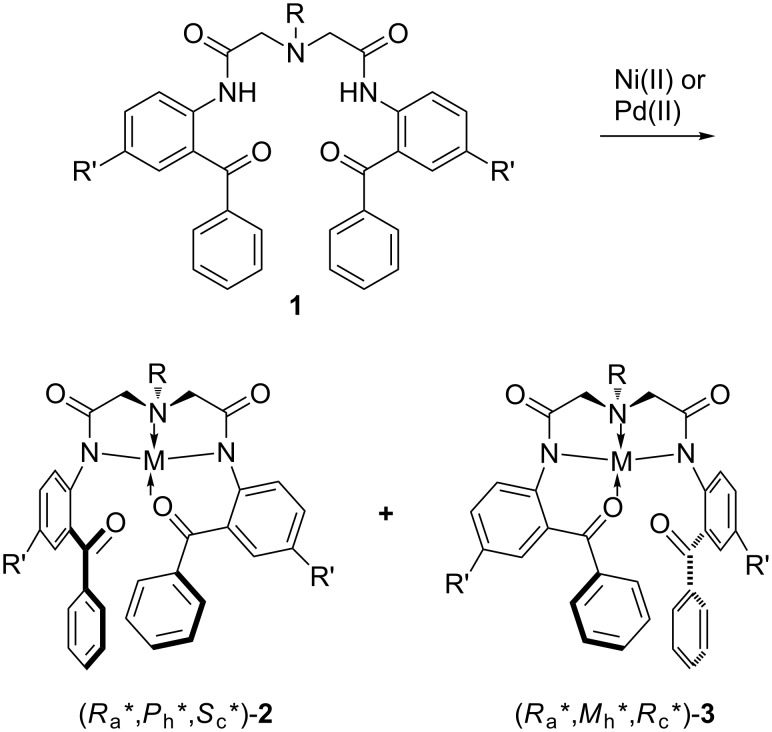
Previous design of diastereomeric molecules **2** and **3** with switchable chirality starting from achiral, *C*_2_-symmetric pentadentate ligands **1**.

Recently, we introduced a new approach to the design of organic molecules with switchable chirality by simple cleavage and formation of metal–ligand coordination bonds, as a potentially useful and conceptually new model for the development of a new generation of organic chiroptical molecular switches [24–25]. Thus, achiral *C*_2_-symmetric pentadentate ligands **1** were able to coordinate with d^8^ metals [Ni(II) or Pd(II)] to form tetra-coordinated diastereomeric complexes (*R*_a_*,*P*_h_*,*S*_c_*)-**2** and (*R*_a_*,*M*_h_*,*R*_c_*)-**3**, which contain at least three elements of chirality, namely stereogenic center, axis and helix (Scheme 1). The structure of these complexes incorporated two five- and one six-membered ring displaying a square-planar geometry. It is worth mentioning that only two out of the four possible diastereomeric complexes were accessed in a highly stereoselective manner, as a result of the intrinsic steric hindrance associated with the design of achiral ligands **1**. In addition, crystallization of the diastereomeric mixtures resulted in the formation of a single diastereomer (*R*_a_*,*P*_h_*,*S*_c_*)-**2** or (*R*_a_*,*M*_h_*,*R*_c_*)-**3** in the solid state, depending on the nature of the chelating metal (Ni or Pd) employed.

**Scheme 2:**
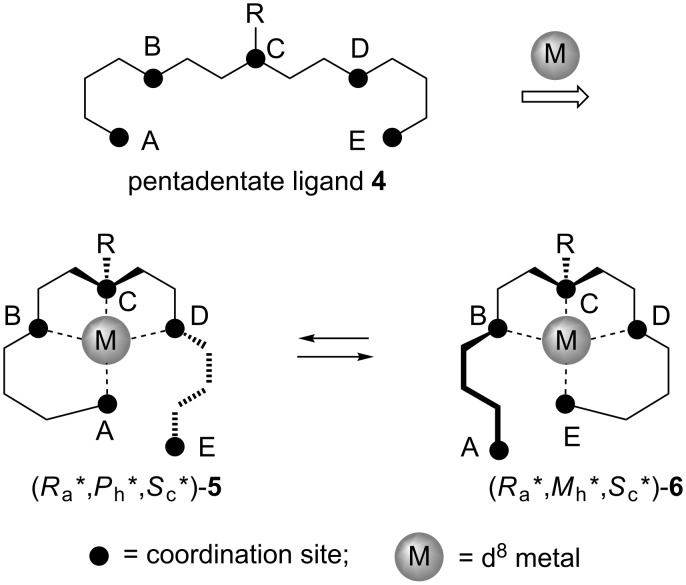
General design of asymmetric pentadentate ligands **4** and chiroptically switchable quasi-diastereomeric molecules **5** and **6**, possessing elements of central, axial and helical chirality.

In this context, we subsequently envisioned the design and synthesis of structurally related complexes in which the chiral switch will be based on trans-coordination of nonsymmetric ligands (Scheme 2).

The major difference of the general design presented in Scheme 2 with the previously reported models (Scheme 1) is that the starting pentadentate ligand **4** is asymmetric and therefore already possesses the stereogenic center at the coordination site **C**. It is assumed that the pentadentate ligand **4** upon coordination with d^8^ metals will give rise to tetra-coordinated square-planar complexes **5** and **6**. The presence of coordinated and noncoordinated groups **A** and **E**, positioned up or down relative to the metal coordination plane, will create an element of axial chirality, directed through the coordination sites **D** in complex **5** and **B** in **6**, and the corresponding adjacent methylenes of the chelating five-membered rings. The second, newly created element of chirality may be provided by steric repulsive interactions in the arms **BA**/**DE**, which will twist the six-membered chelating ring resulting in a screw sense of helical chirality. The transcoordination motion in these complexes is expected to be controlled in a way that noncoordinated sites, i.e., **E** in **5** and **A** in **6**, will move up and down, respectively, resulting in the interconversion of complexes **5** and **6**. This assumed concurrent and unidirectional motion of arms **ED** and **AB** is expected to have the following desired stereochemical consequences: Thus, the axial chirality on the arm **CDE** in **5** will disappear with simultaneous generation of a new chiral axis on the arm **CBA** in **6**, of the same stereochemical sense. Next, this envisioned mode of transcoordination must proceed also with a loss and regeneration of helical chirality, but, in contrast to the loss/regeneration of axial chirality, with the inversion of the stereochemical sense, providing quasi-diastereomeric relationships between complexes **5** and **6** [26].

As one can expect, these desired structural and stereochemical considerations can be seriously compromised by the presence of central chirality in the starting ligand **4**. For instance, application of racemic ligands **4** will give rise to at least four diastereomeric products rendering the designed diastereomeric intertransformation with switchable chirality simply pointless. Furthermore, application of enantiomerically pure ligands **4** can present a problem of mismatched stereochemical preferences between the existing central and newly generated set of axial and helical chirality. On the other hand, if the stereogenic center in ligands **4** is configurationally flexible (can easily undergo (*S*) to (*R*) transformation) one may expect the opposite trend, as the combined stereochemical preferences of axial and helical chirality will impose the corresponding matching configuration of the stereogenic center. Thus, the geometric difference between the designed quasi-diastereomers **5** and **6** is that the noncoordinated arm **CDE** and the substituent R on the stereogenic coordination site **C** in **5** are on the same side of the metal chelation plane, while in structure of **6** the arm **CBA** and the substituent R are on the opposite sides. In contrast, the geometric relations between the off-plane six-membered chelate rings, giving rise to helical chirality, and the substituent R are the same in both quasi-diastereomers **5** and **6**. Considering these geometric features, one may expect that the actual absolute configuration of the stereogenic center **C**, determined by the stereochemical priority of the groups **A** and **E**, has no importance for the quasi-diastereomeric relationships between **5** and **6**. Consequently, there is a good chance that the geometric position of the substituent R, and therefore the relative configuration of the stereogenic center on **C**, may be efficiently controlled by the matching stereochemical preferences between the axial and helical chirality in the quasi-diastereomers **5** and **6**. However, to realize this possibility the stereogenic center **C** in ligands **4** should be configurationally unstable and easily adaptable to the developing stereochemical environment upon its configurational stabilization in complexes **5** and **6**.

## Results and Discussion

**Scheme 3:**
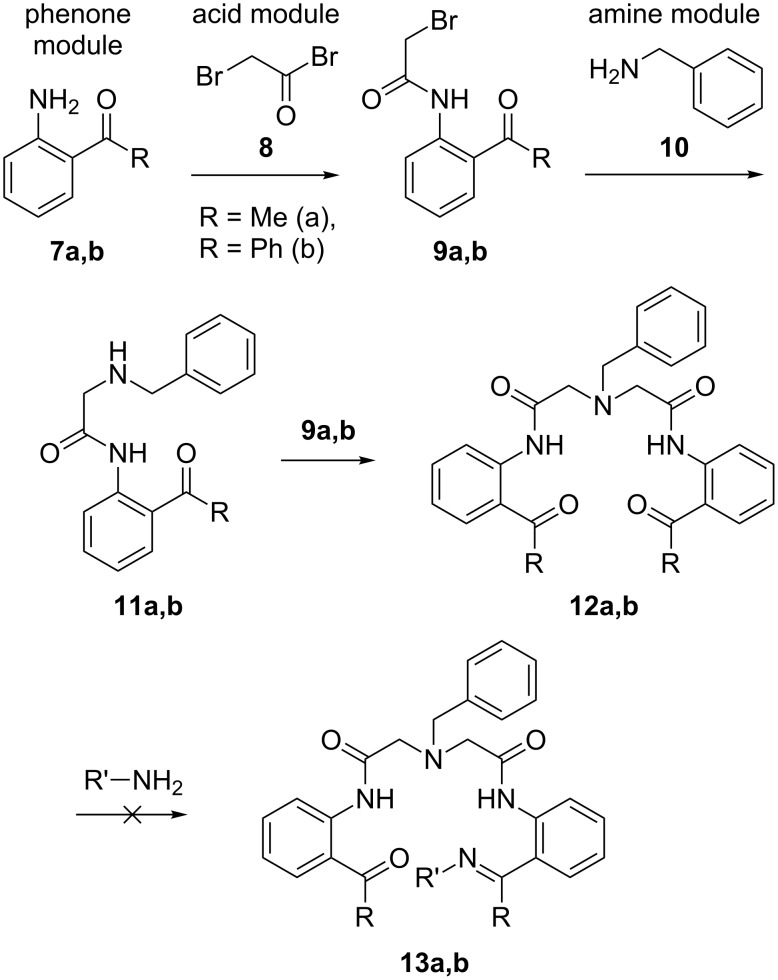
Preparation of imino–carbonyl ligands **13** by desymmetrization of achiral carbonyl–carbonyl ligands **12**.

To build the real molecules to this design, we explored the preparation of imino–carbonyl ligands **13** (Scheme 3) by desymmetrization of achiral carbonyl–carbonyl ligands **12**. Compounds **12** were prepared in the framework of our recently described methodology for applying a new generation of nucleophilic glycine equivalents [27–32] to a general asymmetric synthesis of α-amino acids [33–38]. In this manner, modular assembly of achiral *C*_2_-symmetric pentadentate ligands **12** was carried out by using three inexpensive and readily available starting structural units. First, the “phenone” modules **7** were reacted [39] with “acid” module **8** to form the corresponding amides **9** in quantitative chemical yield. Application of compounds **9** for bis-alkylation of “amine” module **10** was conducted in two separate steps including isolation and purification of the intermediate products **11**. The undesired formation of the corresponding quaternary ammonium salts was not observed [40] and the target ligands **12** were prepared in high overall yield (96%).

Surprisingly, we found that the seemingly simple transformation of **12** to **13** can present a significant synthetic problem. Thus, our attempts to prepare the desired mono-imino ligands **13** from compounds **12**, using one equivalent of various amines, resulted in the formation of complex mixtures of inseparable products. Possible reasons for this outcome are the presence of two potentially reactive amide groups and the multifunctional nature of compounds **12**. In particular, preparation of the most desirable NH-containing (R’ = H) derivatives **13**, by reaction of **12** with ammonia or its derivatives, was not possible at all. Modification of the synthetic scheme with installation of the imino functionality on intermediate stages also gave inacceptable results, probably due to hydrolytic liability of the corresponding imino derivatives of, for instance, compounds **9** and **11**.

While these unexpected synthetic obstacles can, probably, be overcome with a more extensive search for optimized conditions, we decided to take advantage of our experience in the chemistry of Ni(II)-chelated amino acid Schiff bases [41–48] and to develop an indirect approach for the preparation of asymmetric ligands of type **13**. To this goal, we studied the reactions of biscarbonyl compounds **12** with glycine.

**Scheme 4:**
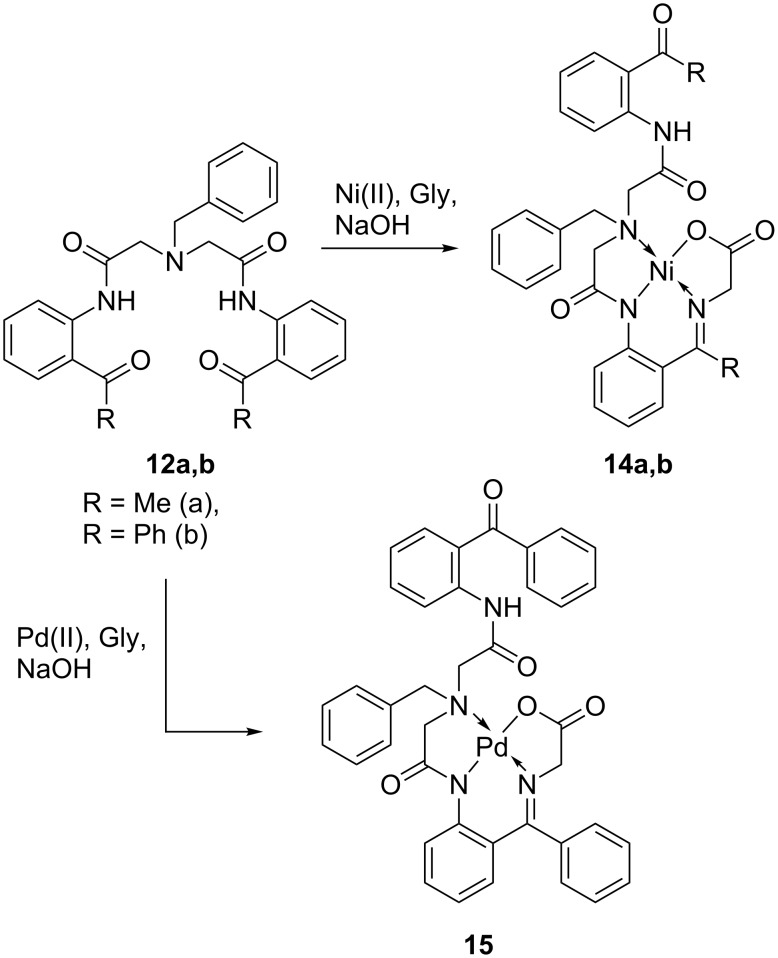
Preparation of complexes **14a**,**b** and **15** by reactions of ligands **12** with glycine.

As shown in Scheme 4, heating of pentadentate ligands **12** in acetonitrile in the presence of a Ni(II) source [NiCl_2_ or Ni(NO_3_)_2_], glycine and a base, lead to the formation of the quite unusual complexes **14**. In a similar manner, by using PdCl_2_ instead of Ni(II), the Pd-chelated complex **15** was obtained. As it follows from these results, only one carbonyl group in the starting ligands **12** was involved in the formation of the corresponding Schiff base with glycine, similar to the reactivity observed for tridentate ligands with Ni(II) [49–53]. In the ^1^H and ^13^C NMR spectra of compounds **14** and **15**, resonances of aromatic and aliphatic hydrogens and carbons appear as sharp peaks indicating that the extra bidentate moiety in complexes **14** and **15** acts merely as a substituent and does not compete for coordination with Ni(II). Complexes **14** are red-colored, **15** is yellow, and both are neutral, highly crystalline compounds (mp > 250 °C), good solubility in organic solvents, and can be easily purified by regular column chromatography on SiO_2_.

**Figure 1:**
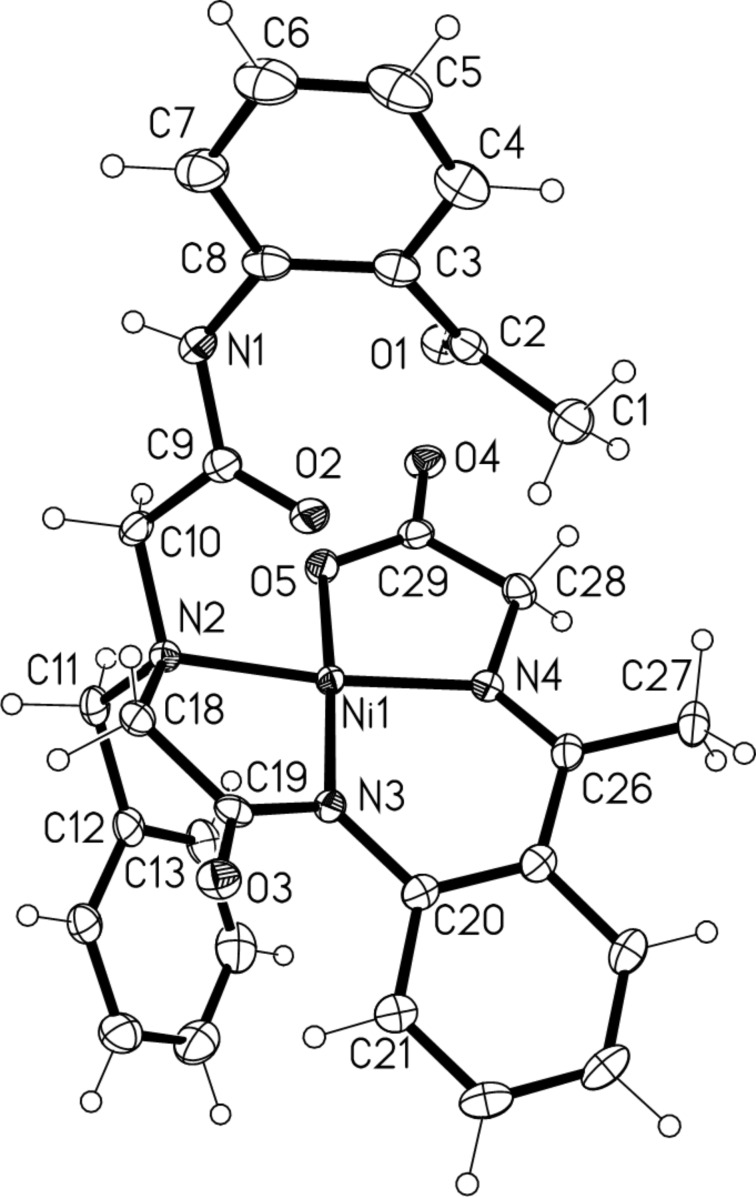
Crystallographic structure of complex **14a**.

To receive more precise data about the structure of complexes **14** and **15** we performed crystallographic analysis of compound **14a** [54] (Figure 1). Quite surprisingly, we found that in the solid state (monoclinic crystal system, space group *P*2_1_/*n*) complex **14a** has three elements of chirality: stereogenic center [N(2)], axis [N(1)–C(8) bond] and helix (off-plane position of the chelate rings). While these three elements of chirality can give up to four possible diastereomeric combinations, only one diastereomer, as a pair of (*R*_c_,*R*_a_,*P*_h_) and (*S*_c_,*S*_a_,*M*_h_) enantiomers, was found in the crystallographic unit cell. This fact clearly suggested that the observed set of stereochemical preferences is a result of a mutual match between these three elements of chirality giving rise to the most stable diastereomeric stereochemistry.

The distances of the Ni–O(5) [1.8651(13) Å] and Ni–N(4) [1.8569(16) Å] bonds of the five-membered chelate ring of the glycine Schiff base fragment were relatively similar, while the bonds Ni–N(3) [1.8417(16) Å] and Ni–N(2) [1.9535(16) Å] were noticeably shorter and longer, respectively. The square-planar structure of compound **14a** is slightly distorted with angles N(4)–Ni–N(2) of 173.48(7)° and N(3)–Ni–O(5) of 175.15(6)°. This deviation from planarity gives compound **14a** a bowl-like shape and renders it inherently chiral. For instance, the torsion angle C(19)–N(3)–C(20)–C(21) of 32.0(3)° sets the element of helical chirality, while the axial chirality is located through the N(1)–C(8) bond with the torsion angle C(9)–N(1)–C(8)–C(7) of −139.5(2)°. The central chirality is located on N(2) as, coordinated to Ni(II), nitrogen is configurationally stable and has four different substituents. As mentioned above, the absorptions of hydrogens and carbons in the NMR spectra, recorded at ambient temperature, are sharp and therefore do not suggest any diastereomeric transformations. Accordingly, one may assume that in CHCl_3_ solution compounds **14** and **15** also exist in one diastereomeric form possibly stabilized by apical coordination of the amidic carbonyl of the pendent side chain (distance of Ni–O(2) = 2.665 Å).

**Scheme 5:**
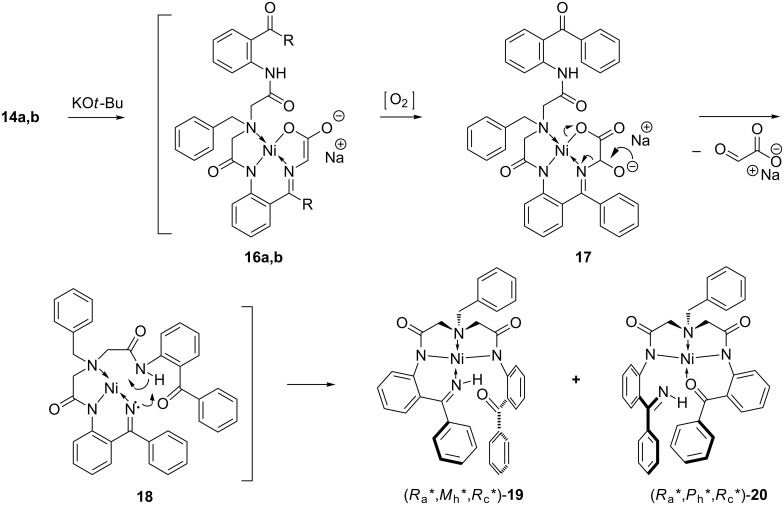
Oxidation of enolates **16** and formation of complexes **19** and **20**.

With complexes **14** and **15** in hand, we next studied the oxidation of **14** as presented in Scheme 5. Formation of the corresponding enolates **16**, in the presence of relatively strong bases (KO*t*-Bu), is an established step in general methods for homologation of the glycine moiety by alkyl halide alkylation [27,50–53], aldol [29,33,36–37] and Michael [28,31,34–35,38–39,43–47] addition reactions. The following oxidation step of the enolates **16** in the presence of atmospheric oxygen, is less studied [48]; however, plausible key transformations can be deduced based on the closely related results on the well-established general oxidation of enolates [55–64] and glycine-derived enolates [65–70], in particular, under similar conditions. Thus, the ionized form of α-hydroxy intermediate **17** can undergo the cleavage of the glycine C–N bond [65,68–70] resulting in the formation of neutral complex **18**. Previously, we demonstrated that in situ formed derivatives of type **18**, without the pendant side chain, undergo further stabilization through dimerization, giving rise to the corresponding binuclear complexes [48]. In this case, the presence of the bidentate substituent allows for a different, intramolecular stabilization mechanism leading to the formation of target complexes **19**/**20**.

It is interesting to note that the acetophenone module-derived compound **14a** gave the expected enolate **16a**; however, its further oxidation lead mostly to decomposition, suggesting higher instability of intermediate derivatives of type **17** and **18** [48]. Also, the benzophenone-derived, Pd(II)-containing analogue **15** (Scheme 4) failed to produce the target complexes of type **19**/**20**, pointing to some limitation of this synthetic alternative. Nevertheless, this oxidative pathway allowed for convenient preparation of otherwise inaccessible complexes **19**/**20** formally derived from asymmetric carbonyl/imine ligands **13** (Scheme 3) and Ni(II). In contrast to typical Ni(II) complexes, such as **14a,b**, compounds **19**/**20** display an unusual dark-brown color. Nevertheless, their high solubility in polar organic solvents allows for their straightforward purification by column chromatography. Initial assignment of the structures of compounds **19**/**20** by NMR was not possible because of the very fast exchange between Ni–O=C and Ni–NH=C chelation. Thus, due to fast interconversion between compounds **19** and **20**, only broad signals were observed in the ^1^H NMR spectra recorded at ambient temperature. However, variable temperature experiments (−50 °C) produced a notable sharpening in both aromatic and aliphatic protons. Ultimately, elucidation of their structure was carried out by X-ray analysis, which was feasible due to their high crystallinity [54].

**Figure 2:**
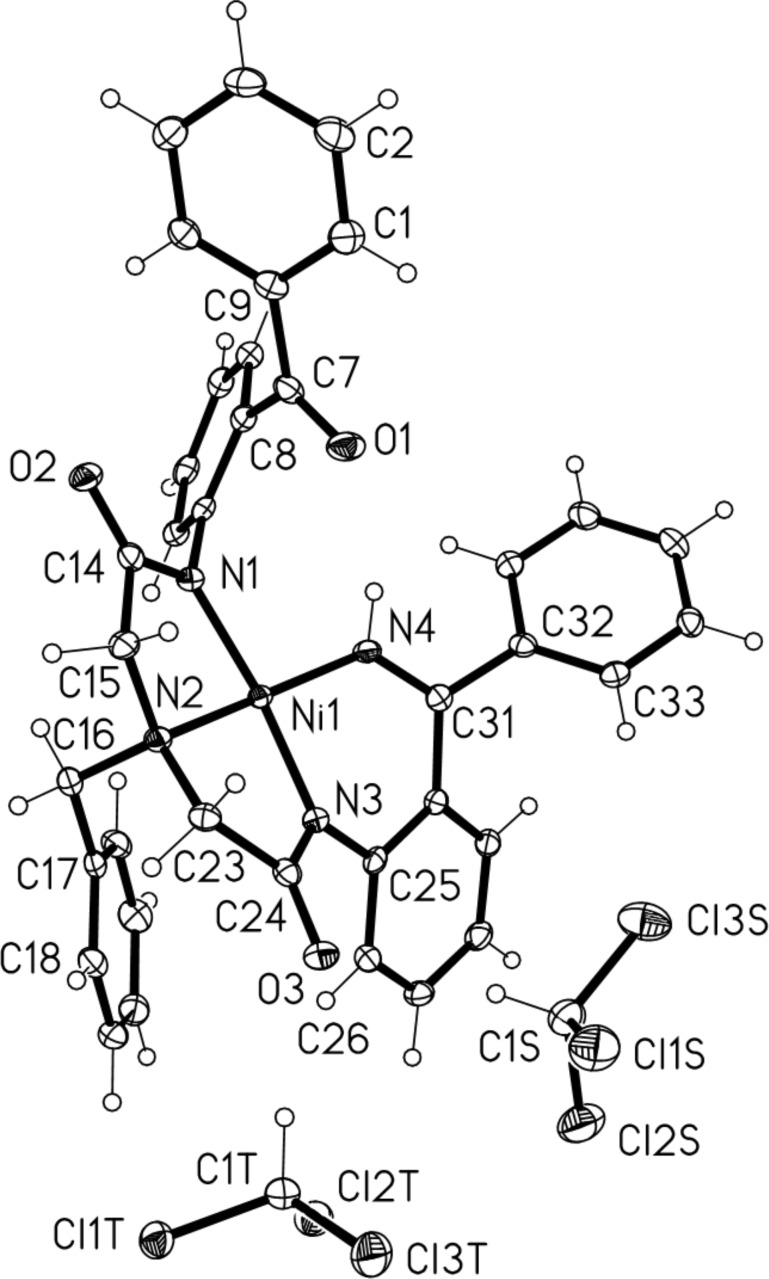
Crystallographic structure of complex **19**.

We found that in the solid state, compounds **19** and **20** exist as the sole NH–Ni coordinated quasi-diastereomer (*R*_a_*,*M*_h_*,*R*_c_*)-**19** (Figure 2), containing a pair of (*R*_a_,*M*_h_,*R*_c_) and (*S*_a_,*P*_h_,*S*_c_) enantiomers in the crystallographic unit cell (triclinic, 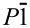 space group). Surprisingly, the distance of the Ni–NH bond was found to be the shortest [1.8518(15) Å] in the Ni(II)-coordinated plane, followed by the Ni–N(3) [1.8524(14) Å] of the same five-membered chelate ring. Two other Ni–N(1) [1.8760(14) Å] and Ni(1)–N(2) [1.9076(15) Å] were noticeably longer. The square-planar coordination plane in compound **19** was substantially more distorted, as compared with complex **14a** or carbonyl-coordinated analogues **2** and **3** [24–25] (Scheme 1), with the angles N(3)–Ni–N(1) of 171.94(6)° and N(4)–Ni–N(2) of 169.80(6)° rendering it inherently chiral. Consequently, the torsion angle C(24)–N(3)–C(25)–C(26) of 34.4(2)° gives rise to an element of helical chirality in compound **19**. The element of axial chirality is generated by the restricted rotation around the N(1)–C(13) bond with the torsion angle C(14)–N(1)–C(13)–C(12) of −119.59(18)°. The third element of chirality, a stereogenic center, is located on N(2).

## Conclusion

In conclusion, we have shown that the newly prepared Ni(II) complexes derived from asymmetric, pentadentate ligands exist as quasi-diastereomers, generating two new elements of chirality (stereogenic axis and helix) to the pre-existing configurationally stable stereogenic nitrogen. The stereoselective formation of only two, (*R*_a_*,*M*_h_*,*R*_c_*) and (*R*_a_*,*P*_h_*,*R*_c_*), out of the four possible stereochemical combinations is most remarkable, arising due to the highly stereocongested structural characteristics of these Ni(II) complexes. The quasi-diastereomeric transformation between products (*R*_a_*,*M*_h_*,*R*_c_*) and (*R*_a_*,*P*_h_*,*R*_c_*) occurs by intramolecular transcoordination of Ni–NH and Ni–O bonds. This transcoordination results in the loss and regeneration of axial and helical chirality with retention and inversion, respectively, of the stereochemical sense. The stereogenic center in this case remains unchanged. As it follows from the crystallographic studies, in the solid state there is an obvious preference for Ni–NH over Ni–O coordination. The flexibility of this modular design, in which the three major “phenone”, “acid” and “amine” modules can be selected by using many structural modifications, provides a very practical tool for the preparation of a wide variety of Ni(II) complexes, which may result in a higher sensitivity to chemical properties such as temperature, solvent or pH of the reaction medium.

## Experimental

### General

^1^H and ^13^C NMR were performed on Varian Unity-300 (299.94 MHz) and Gemini-200 (199.98 MHz) spectrometers with TMS and CDCl_3_ as internal standards. High-resolution mass spectra (HRMS) were recorded on a JEOL HX110A instrument. Optical rotations were measured on a JASCO P-1010 polarimeter. Melting points (mp) are uncorrected and were obtained in open capillaries. All reagents and solvents, unless otherwise stated, are commercially available and were used as received. Unless otherwise stated, yields refer to isolated yields of products of greater than 95% purity as estimated by ^1^H and ^13^C NMR spectrometry. All new compounds were characterized by ^1^H, ^13^C NMR and HRMS. For preparation of **12a,b** see [25,39].

#### Synthesis of complexes 14a,b and 15

**General procedure.** To a suspension of compound **12**, glycine (5 equiv), and MX_2_ (NiCl_2_, Ni(NO_3_)_2_ or PdCl_2_, 2 equiv) in MeOH, NaOH (7 equiv) was added, and the reaction mixture was stirred at 60–70 °C for 4 h. Then, the reaction mixture was poured over a slurry of ice and 5% AcOH. After complete precipitation, the solid was filtered, washed with water, and dried. The product was purified by silica-gel column chromatography (CHCl_3_/acetone = 4:1).

**Complex 14a** (46% yield). Mp 277.2 °C (dec); ^1^H NMR (300 MHz, CDCl_3_) δ 2.34 (s, 3H), 2.68 (s, 3H), 2.89 (d, *J* = 15.4 Hz, 1H), 3.30 (d, *J* = 16.5 Hz, 1H), 3.83 (d, *J* = 15.4 Hz, 1H), 3.85 (d, *J* = 16.2 Hz, 1H), 3.86 (d, *J* = 18.8 Hz, 1H), 3.94 (d, *J* = 12.6 Hz, 1H), 4.43 (d, *J* = 19.0 Hz, 1H), 4.54 (d, *J* = 12.8 Hz, 1H), 6.92 (ddd, *J* = 8.30, 7.13, 1.18 Hz, 1H), 7.16–7.24 (m, 2H), 7.30–7.36 (m, 1H), 7.37–7.46 (m, 2H), 7.50 (dd, *J* = 8.30, 1.46 Hz, 1H), 7.64 (ddd, *J* = 8.69, 7.13, 1.56 Hz, 1H), 7.93 (dd, *J* = 8.01, 1.56 Hz, 1H), 8.06 (dd, *J* = 8.59, 1.17 Hz, 1H), 8.09–8.15 (m, 2H), 8.97 (dd, *J* = 8.49, 1.17 Hz, 1H); ^13^C NMR (75 MHz, CDCl_3_) δ 18.9, 28.5, 59.8, 60.0, 60.9, 64.4, 121.2, 121.3, 122.1, 123.3, 124.8, 126.8, 128.7, 128.9, 129.4, 131.4, 131.5, 131.7, 132.1, 135.2, 139.9, 141.4, 166.7, 168.8, 176.1, 176.8, 203.2; HRMS–ESI (*m*/*z*): [M + Na]^+^ calcd for C_29_H_28_N_4_NaNiO_5_, 593.1311; found, 593.1321.

**Complex 14b** (83% yield). Mp 259.3 °C (dec); ^1^H NMR (300 MHz, CDCl_3_) δ 2.83 (d, *J* = 16.5 Hz, 1H), 3.37 (d, *J* = 16.4 Hz, 1H), 3.65 (d, *J* = 19.8 Hz, 1H), 3.84 (d, *J* = 12.2 Hz, 1H), 3.88 (d, *J* = 13.2 Hz, 1H), 3.89 (d, *J* = 19.8 Hz, 1H), 4.14 (d, *J* = 12.8 Hz, 1H), 4.70 (d, *J* = 13.1 Hz, 1H), 6.72 (ddd, *J* = 8.30, 6.84, 1.27 Hz, 1H), 6.84 (dd, *J* = 8.20, 1.76 Hz, 1H), 7.00–7.74 (m, 17H), 7.90–8.00 (m, 2H), 8.47 (d, *J* = 8.79 Hz, 1H), 8.83 (d, *J* = 7.91 Hz, 1H), 11.1 (bs, 1H); ^13^C NMR (75 MHz, CDCl_3_) δ 59.0, 60.5, 61.2, 63.9, 120.7, 122.6, 123.3, 124.2, 125.1, 125.5, 125.9, 126.3, 128.2, 128.9, 129.1, 129.4, 129.4, 129.6, 129.8, 131.1, 132.0, 132.1, 132.5, 133.0, 133.1, 133.9, 134.7, 138.1, 138.7, 142.4, 166.5, 171.0, 176.7, 177.1, 199.2; HRMS–ESI (*m*/*z*): [M + H]^+^ calcd for C_39_H_33_N_4_NiO_5_, 695.1804; found, 695.1801.

**Complex 15** (86% yield). Mp 253.5 °C (dec); ^1^H NMR (300 MHz, CDCl_3_): δ 3.77 (d, *J* = 15.8 Hz, 1H), 4.11 (d, *J* = 11.6 Hz, 1H), 4.12 (s, 2H), 4.18 (d, *J* = 11.1 Hz, 1H), 4.30 (d, *J* = 16.4 Hz, 1H), 4.46 (s, 2H), 6.78 (ddd, *J* = 8.20, 7.03, 1.17 Hz, 1H), 6.92–7.01 (m, 2H), 7.13–7.22 (m, 2H), 7.25–7.88 (m, 16H), 8.47 (dd, *J* = 8.79, 1.18 Hz, 1H), 8.58 (dd, *J* = 8.50, 1.18 Hz, 1H), 11.0 (bs, 1H); ^13^C NMR (75 MHz, CDCl_3_) δ 61.1, 63.1, 64.0, 65.3, 121.2, 122.4, 123.4, 123.6, 125.0, 126.0, 126.2, 126.4, 128.3, 128.8, 129.4, 129.5, 129.7, 129.7, 129.9, 131.5, 132.3, 132.7, 133.0, 133.1, 133.8, 134.2, 134.9, 137.9, 138.6, 142.2, 165.0, 169.2, 176.6, 177.8, 198.9; HRMS–ESI (*m*/*z*): [M + H]^+^ calcd for C_39_H_33_N_4_O_5_Pd, 743.1436; found, 743.1501.

**Complexes 19/20.** To a solution of **14b** (1 equiv) in CH_3_CN, was added KO*t*-Bu (4 equiv) at rt and the reaction mixture was stirred at rt under aerobic conditions until the starting compound was consumed completely, as confirmed by TLC. After evaporation of the solvent, water and a calculated amount of 5% AcOH aq. (4 equiv) was added and extracted with CH_2_Cl_2_ three times. The combined organic layers were dried over MgSO_4_, and the product was purified on a short-path flash silica-gel column. Due to the fast exchange between complexes **19/20**, NMR spectra were not properly recorded. Mp 264.5 °C (dec); HRMS–ESI (*m*/*z*): [M + Na]^+^ calcd for C_37_H_30_N_4_NaNiO_3_, 659.1569; found, 659.1547.

## Supporting Information

File 1 NMR spectra of compounds **14b** and **19/20**.
