# Supplementary material for: Design and synthesis of quasi-diastereomeric molecules with unchanging central, regenerating axial and switchable helical chirality via cleavage and formation of Ni(II)–O and Ni(II)–N coordination bonds
Source: Beilstein J Org Chem. 2012 Nov 13;8:1920–8. doi: 10.3762/bjoc.8.223 (PMC3511032; doi:10.3762/bjoc.8.223)

Supporting Information

for

Design and synthesis of quasi-diastereomeric molecules with unchanging central, regenerating axial and switchable helical chirality via cleavage and formation of Ni(II)–O and Ni(II)–N coordination bonds

Vadim A. Soloshonok*[1,2], José Luis Aceña[1], Hisanori Ueki[3] and Jianlin Han[4]

Address: [1]Department of Organic Chemistry I, Faculty of Chemistry, University of the Basque Country, 20018 San Sebastián, Spain, [2]IKERBASQUE, Basque Foundation for Science, 48011 Bilbao, Spain, [3]International Center for Materials Nanoarchitectonics (MANA), National Institute for Materials Science (NIMS), 1-1, Namiki, Tsukuba, Ibaraki 305-0044, Japan and [4]School of Chemistry and Chemical Engineering, Nanjing University, Nanjing, 210093, China

Email: Vadim A. Soloshonok* - vadym.soloshonok@ehu.es

* Corresponding author

NMR spectra of compounds 14b and 19/20

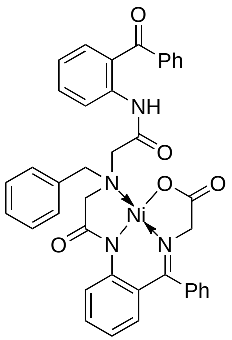

14b

(300 MHz, $CDCl_3$)

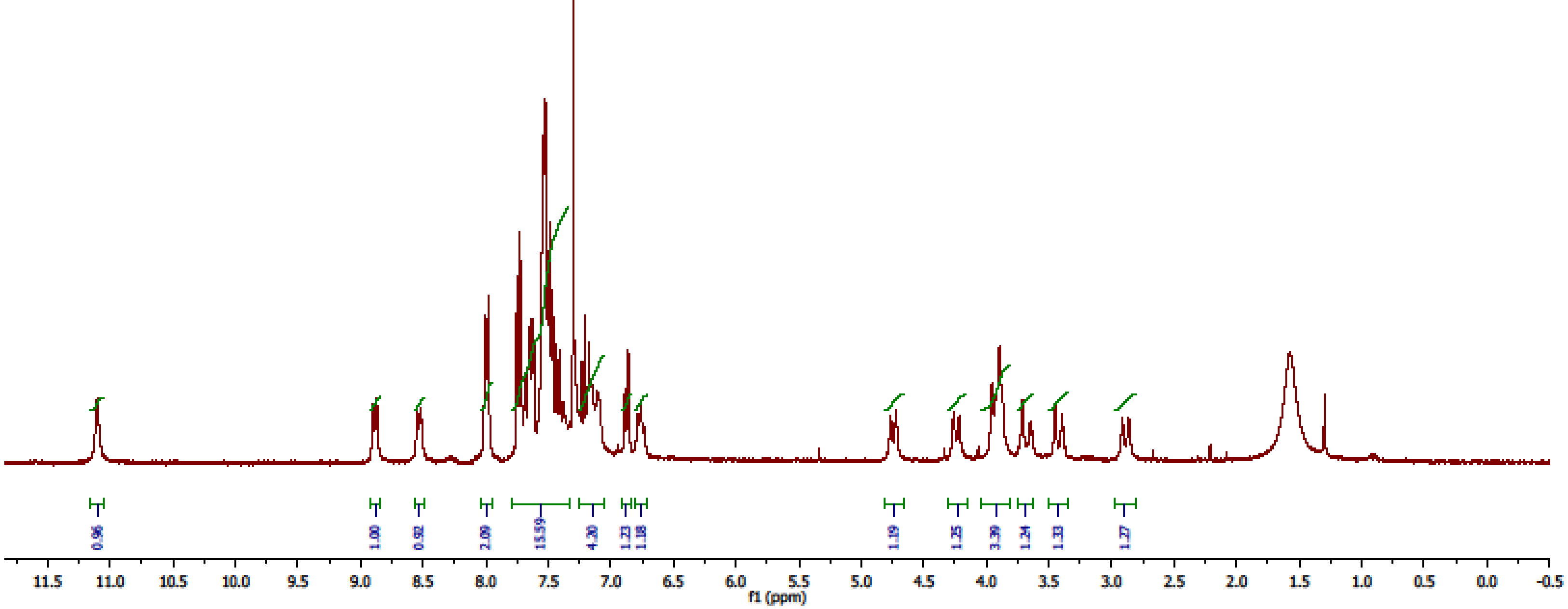

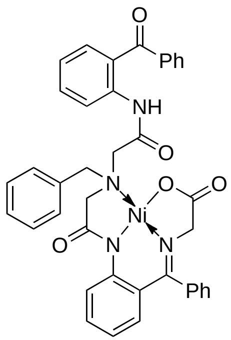

14b

(75 MHz, $CDCl_3$)

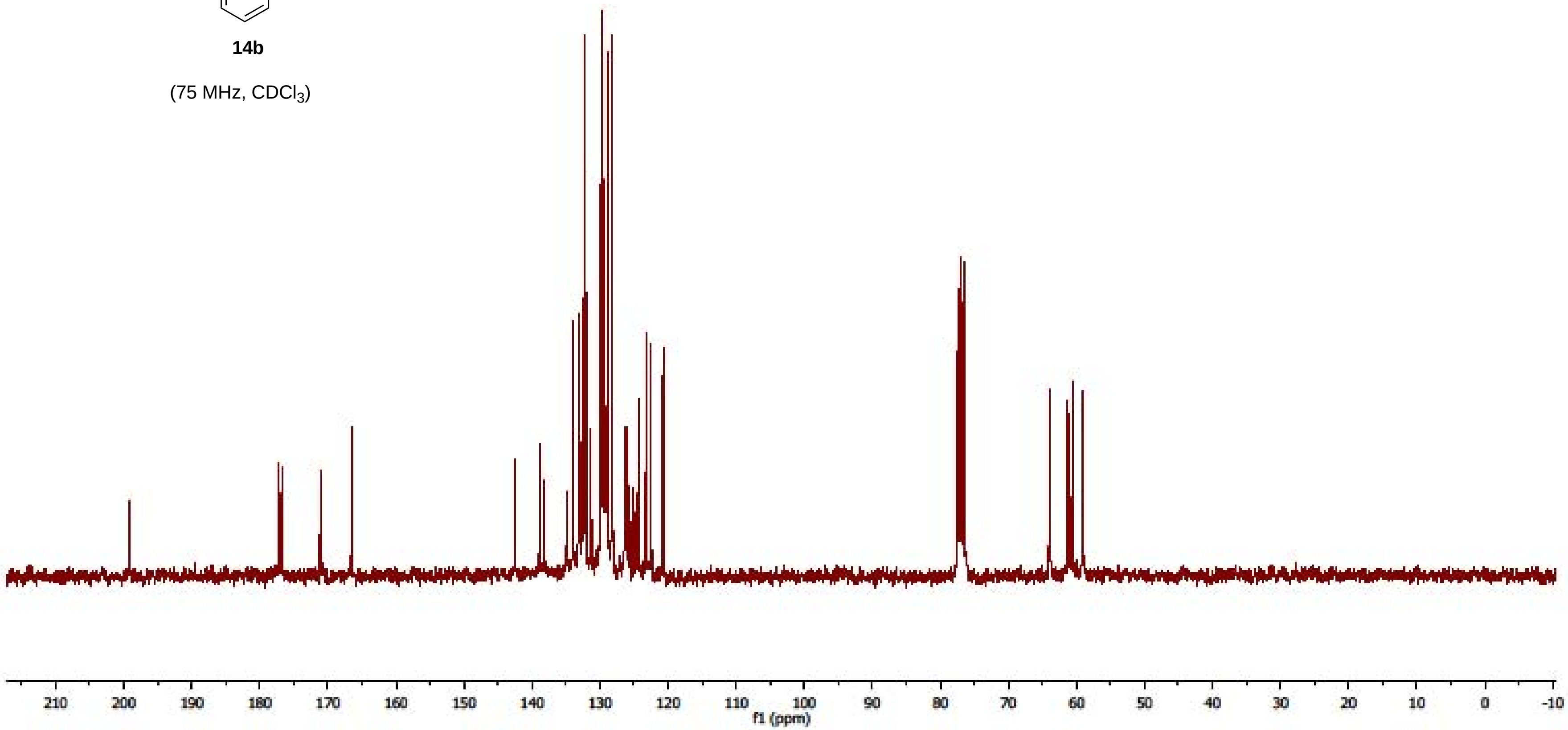

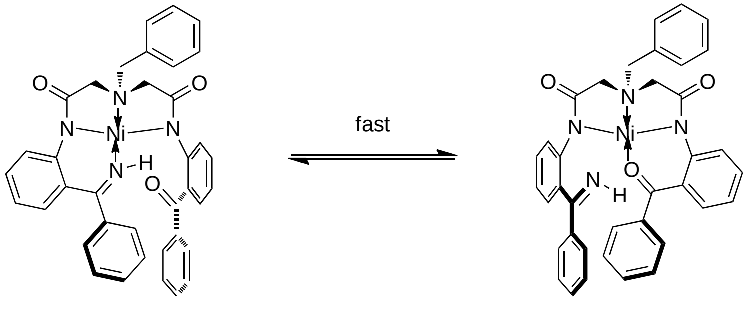

19 **20**

(300 MHz, $CDCl_3$, 25 ºC)

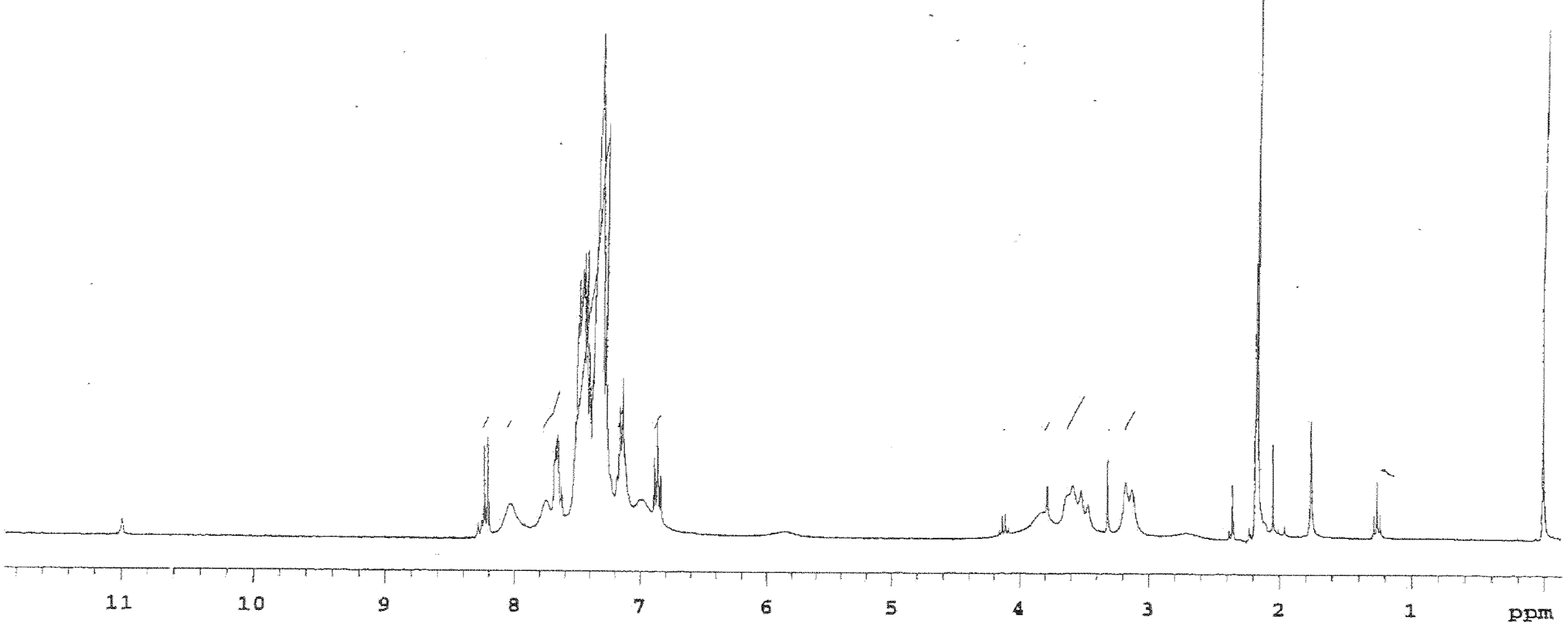

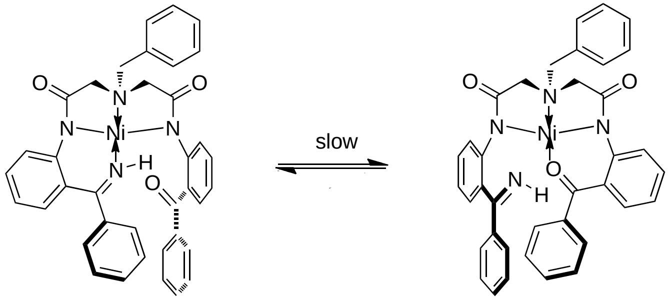

19 **20**

(300 MHz, $CDCl_3$, –50 ºC)

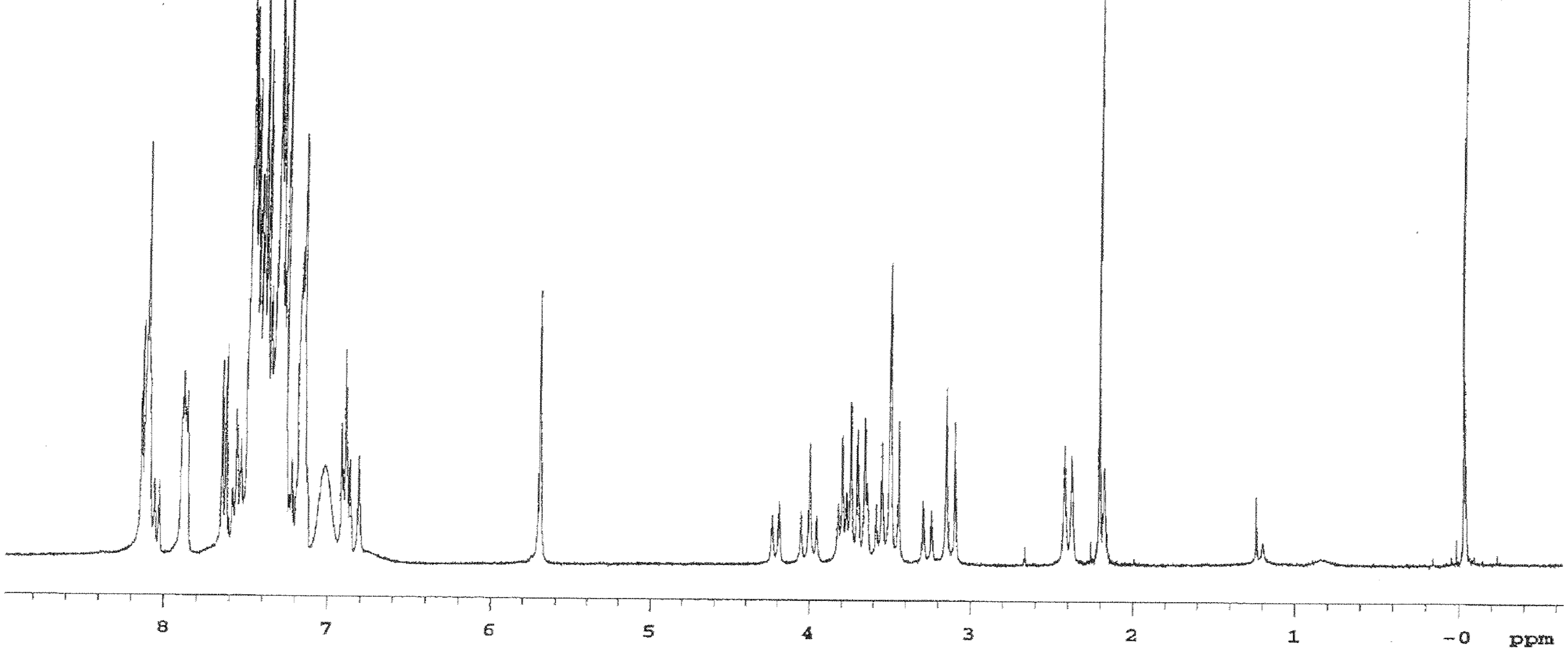

Supplement: File 1 — NMR spectra of compounds 14b and 19/20. [file Beilstein_J_Org_Chem-08-1920-s001.pdf]
